# Complete genome sequence of the sugarcane nitrogen-fixing endophyte *Gluconacetobacter diazotrophicus *Pal5

**DOI:** 10.1186/1471-2164-10-450

**Published:** 2009-09-23

**Authors:** Marcelo Bertalan, Rodolpho Albano, Vânia de Pádua, Luc Rouws, Cristian Rojas, Adriana Hemerly, Kátia Teixeira, Stefan Schwab, Jean Araujo, André Oliveira, Leonardo França, Viviane Magalhães, Sylvia Alquéres, Alexander Cardoso, Wellington Almeida, Marcio Martins Loureiro, Eduardo Nogueira, Daniela Cidade, Denise Oliveira, Tatiana Simão, Jacyara Macedo, Ana Valadão, Marcela Dreschsel, Flávia Freitas, Marcia Vidal, Helma Guedes, Elisete Rodrigues, Carlos Meneses, Paulo Brioso, Luciana Pozzer, Daniel Figueiredo, Helena Montano, Jadier Junior, Gonçalo de Souza Filho, Victor Martin Quintana Flores, Beatriz Ferreira, Alan Branco, Paula Gonzalez, Heloisa Guillobel, Melissa Lemos, Luiz Seibel, José Macedo, Marcio Alves-Ferreira, Gilberto Sachetto-Martins, Ana Coelho, Eidy Santos, Gilda Amaral, Anna Neves, Ana Beatriz Pacheco, Daniela Carvalho, Letícia Lery, Paulo Bisch, Shaila C Rössle, Turán Ürményi, Alessandra Rael Pereira, Rosane Silva, Edson Rondinelli, Wanda von Krüger, Orlando Martins, José Ivo Baldani, Paulo CG Ferreira

**Affiliations:** 1 Instituto de Bioquímica Médica, UFRJ, CCS, Bloco D, subssolo 21491-590 Rio de Janeiro, Brazil; 2 Departamento de Bioquímica, Instituto de Biologia Roberto Alcântara Gomes, UERJ, Blv 28 de Setembro, 87, fundos, 4 andar, Vila Isabel, Rio de Janeiro, RJ 20551-013, Brazil; 3 Laboratório de Tecnologia em Bioquímica e Microscopia, Centro Universitário Estadual da Zona Oeste, Rio de Janeiro, 23070-200, Brazil; 4 Embrapa Agrobiologia BR465, Km 07 Seropédica Rio de Janeiro, 23851-970, Brazil; 5 Instituto de Biologia, Departamento de Entomologia e Fitopatologia, Universidade Federal Rural do Rio de Janeiro Cx Postal 74585/BR 465, KM 07, Seropédica, RJ 23851-970, Brazil; 6 Lab. Biotecnologia- Centro de Biociências e Biotecnologia Universidade Estadual do Norte Fluminense- Av. Alberto Lamego 2000 Campos dos Goytacazes RJ 28013-620, Brazil; 7 Departamento de Biofísica e Biometria, Instituto de Biologia Roberto Alcântara Gomes UERJ, Blv 28 de Setembro, 87, fundos, 4 andar, Vila Isabel, Rio de Janeiro, RJ 20551-013, Brazil; 8 Departamento de Informática - Pontifícia Universidade Católica do Rio de Janeiro Rua Marquês de S. Vicente, 225, Rio de Janeiro 22453-900, Brazil; 9 Departamento de Genética, Instituto de Biologia, Universidade Federal do Rio de Janeiro, Cx Postal 68011, Rio de Janeiro, RJ 21941-617, Brazil; 10 Instituto de Biofísica Carlos Chagas Filho Universidade Federal do Rio de Janeiro, CCS, Cidade Universitária, Rio de Janeiro, RJ21.949-900, Brazil; 11 Laboratório de Biologia Molecular, Departamento de Genética e Biologia Molecular, Universidade Federal do Estado do Rio de Janeiro, Rio de Janeiro, RJ 22290-240, Brazil; 12 Laboratório de Biologia Molecular de Plantas, Instituto de Pesquisas do Jardim, Botânico do Rio de Janeiro, 22460-030 Rio de Janeiro, RJ, Brazil

## Abstract

**Background:**

*Gluconacetobacter diazotrophicus *Pal5 is an endophytic diazotrophic bacterium that lives in association with sugarcane plants. It has important biotechnological features such as nitrogen fixation, plant growth promotion, sugar metabolism pathways, secretion of organic acids, synthesis of auxin and the occurrence of bacteriocins.

**Results:**

*Gluconacetobacter diazotrophicus *Pal5 is the third diazotrophic endophytic bacterium to be completely sequenced. Its genome is composed of a 3.9 Mb chromosome and 2 plasmids of 16.6 and 38.8 kb, respectively. We annotated 3,938 coding sequences which reveal several characteristics related to the endophytic lifestyle such as nitrogen fixation, plant growth promotion, sugar metabolism, transport systems, synthesis of auxin and the occurrence of bacteriocins. Genomic analysis identified a core component of 894 genes shared with phylogenetically related bacteria. Gene clusters for *gum*-like polysaccharide biosynthesis, *tad *pilus, quorum sensing, for modulation of plant growth by indole acetic acid and mechanisms involved in tolerance to acidic conditions were identified and may be related to the sugarcane endophytic and plant-growth promoting traits of *G. diazotrophicus*. An accessory component of at least 851 genes distributed in genome islands was identified, and was most likely acquired by horizontal gene transfer. This portion of the genome has likely contributed to adaptation to the plant habitat.

**Conclusion:**

The genome data offer an important resource of information that can be used to manipulate plant/bacterium interactions with the aim of improving sugarcane crop production and other biotechnological applications.

## Background

In recent years, concerns about fossil fuel supplies and prices have motivated the search for renewable biofuels. With the existing technologies and current costs of fuel transportation, ethanol from sugarcane is the most viable alternative. In some countries, including Brazil, sugarcane is planted with low amounts of nitrogen fertilizers and there is evidence that the use of low levels of nitrogen can be compensated by Biological Nitrogen Fixation (BNF) [1]. Although several organisms are capable of contributing to BNF, it has been shown that the diazotroph Alphaproteobacteria *Gluconacetobacter diazotrophicus *Pal5 (GDI), present in large numbers in the intercellular space of sugarcane roots, stem and leaves, fixes N_2 _inside sugarcane plants, without causing apparent disease [2,3]. Remarkable characteristics of this bacterium are the acid tolerance, the inability to use nitrate as sole nitrogen source and the ability to fix nitrogen in the presence of ammonium in medium with high sugar concentration [2]. Although isolation of GDI from the sugarcane rhizosphere has been reported [4], its poor survival soil and complete absence in soil samples collected between sugarcane rows strongly support the endophytic nature of this nitrogen fixing bacterium [5-7]. In addition to BNF, GDI has other characteristics that contribute to its biotechnological importance: 1-) A *nif*- mutant enhances plant growth, particularly in roots, indicating that GDI secretes plant growth-promoting substances [8]; 2-) It produces a lysozyme-like bacteriocin that inhibits the growth of the sugarcane pathogen *Xanthomonas albilineans *[9]; 3-) It has antifungal activity against *Fusarium sp*. and *Helminthosporium carbonum *[10]; 4-) GDI promotes an increase in the solubility of phosphate and zinc [11]. Besides its biotechnological features, the genome is especially interesting be-cause is the third diazotrophic endophytic bacteria to be completed sequenced. The first two diazotrophic endophytes to be sequenced, *Azoarcus sp*. strain BH72 [12] and *Klebsiella pneumoniae *342 [13], belong to the Betaproteobacteria and Gammaproteobacteria classes, respectively. Thus, the genome of GDI is the first to be completely sequence from Alphaproteobacteria class. Here we report the complete genome sequence of the *G. diazotrophicus *strain Pal5. Sequence analyzes show the existence of a large accessory genome, probably originated by extensive Horizontal Gene Transfer (HGT). Moreover, experimental results reveal differences in Genomic Islands (GI) among *G. diazotrophicus *strains. The knowledge of the metabolic routes, organization and regulation of genes involved in nitrogen fixation, establishment of successful plant association and other processes should allow a better understanding of the role played by this bacterium in plant-bacteria interaction.

## Results

### Overview of the *G. diazotrophicus *PAL5 genome

The complete genome of GDI is composed of one circular chromosome of 3,944,163 base pairs (bp) with an average G+C content of 66.19%, and two plasmids of 38,818 and 16,610 bp, respectively (table 1). The circular chromosome has a total of 3,864 putative coding sequences (CDS), with an overall coding capacity of 90.67%. Among the predicted genes, 2,861 were assigned a putative function, and 1,077 encode hypothetical proteins. Regarding noncoding RNA genes, 12 rRNAs (four rRNA operons) and 55 tRNAs were identified. The larger plasmid (pGD01) has 53 CDS; approximately 70% encode hypothetical or conserved hypothetical proteins and five encode proteins involved in plasmid-related functions. The remaining 11 CDS encode putative components of the Type IV secretion system (T4SS). The small plasmid (pGD02) has 21 CDS, and around 50% are hypothetical proteins.

**Table 1:** General features of the *G. diazotrophicus *PAL5 genome.

**Features**	
Size, bp	3,999,591
G+C content, %	66%
Coding sequences	3,938
Functional assigned	2,861
Insertion Sequences (IS)	223
Pseudo genes	83
Conserved and hypothetical proteins	1,077
% of the genome coding	90
Average length, bp	947
%ATG initiation codons	2,809
%GTG initiation codons	681
%TTG initiation codons	440

RNA elements	

rRNA	4 × (16S-23S-5S)
tRNAs	55

Although today the genome databases have more than 800 complete microbial genomes, only nine are endophytes (*Azoarcus sp*. BH72, *Burkholderia phytofirmans *PsJN, *Enterobacter sp*. 638, *Methylobacterium populi *BJ001, *Pseudomonas putida *W619, *Serratia proteamaculans *568, *Klebsiella pneumoniae *342, *Stenotrophomonas maltophilia *R551-3 and *Gluconacetobacter diazotrophicus *Pal5) [14]. The complete genomes of endophytic bacteria reveal remarkably few mobile elements in its genome (Additional file 1), an observation that led to the proposal that this could denote an adaptation to a more stable life style [12]. In contrast, GDI contains 190 transposable elements, more than any other endophytic bacteria (Additional file 1). The large number of mobile elements could be a signature of a recent evolutionary bottleneck and consequent relaxation of selection, perhaps due to a recent change in niche [15]. Alternatively, because GDI is found in low frequency at the rhizosphere, the transposable elements could have been acquired from other bacteria inhabiting the same environment. In order to identify possible specific characteristics of the genome, the Predicted Highly Express Genes (PHX) genes were identified [16]. PHX analysis identified 658 CDS (17% of the chromosome) in GDI with E(g) (general expression level) > 1,0. Combining this information with the proteomic results [17], which sequenced peptides from 541 genes, we identified 318 of these genes PHX. As expected, ribosomal proteins, translation/transcription factors and chaperone/degradation genes are among the top 30 E(g) values within the 318 CDS, (Additional file 2). However, some unexpected CDS also appear as PHX. For instance, there are 50 transporter proteins or transporter-related proteins with high E(g) value, of which 27 are putative ABC transporter proteins and six are putative TonB-dependent receptors. The genome has two ammonium transporter proteins (GDI0706 and GDI2352) and both with high E(g) values. Two other proteins related to ammonium metabolism are also PHX: a putative glutamate-ammonia-ligase adenyltransferase (GDI3425) and a putative histidine-ammonia-lyase (GDI0550). This finding is consistent with the fact that ammonium is the preferred nitrogen source for GDI when it is available.

### Core and accessory regions

**Figure 1:**
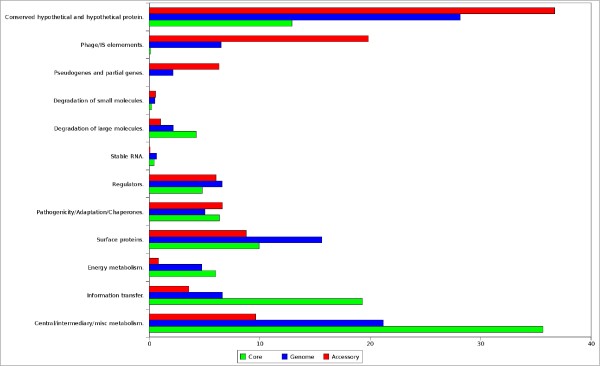
**Distribution of gene class by groups**. Percentage of gene class in three groups: Whole genome (blue), core regions (green) and accessories regions (red). The group energy metabolism includes glycolysis, electron transport. Information transfer includes transcription, translation and DNA/RNA modification. Surface class includes inner and outer membrane, secreted proteins, and lipopolysaccharides.

**Figure 2:**
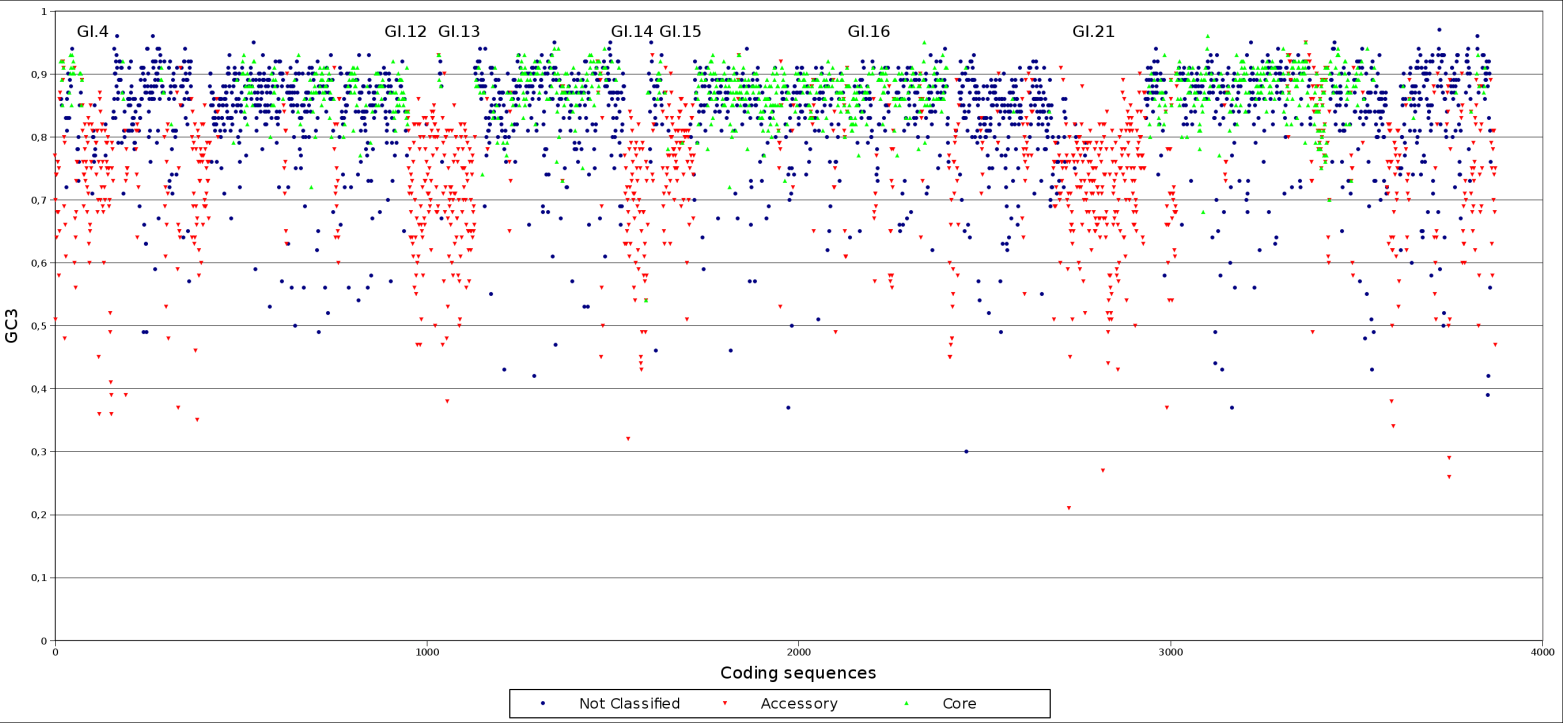
**GC3 analysis of all genes in the chromosome**. Each spot represents a gene in the chromosome. In red are the genes that were classified as accessories by the IVOM method. In green are the genes classified as core by quartops analysis. In blue are the genes that were not classified as core or accessories.

**Figure 3:**
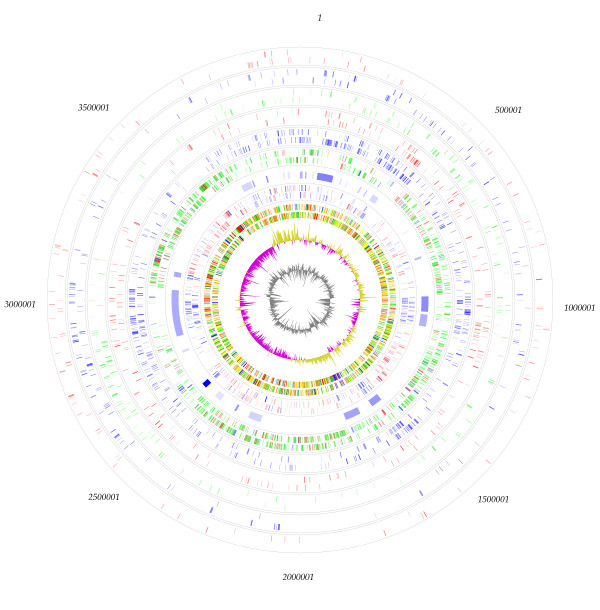
**Circular representation of *G. diazotrophicus *PAL5 chromosome**. From inside to outside. 1-) GC Content. 2-) GC Skew. 3-) Annotation, colors defined by class, see Methods. 4-) Predicted Highly Expressed genes; in blue genes classified as "Alien" and in red genes classified as putative highly expressed. 5-) Accessory regions determined by GC3 and IVOM. 6-) Reciprocal best hits results, in green from *G. oxydans *621H, in blue genes from *A. cryptum *JF-5 and in red genes from *G. bethesdensis *CGDNIH. 7-) Reciprocal Best Hits (RBH) with all complete genomes from the order rhizobiales. 8-) RBH with all other complete genomes from Alphaproteobacteria class; 9-) RBH with all complete genomes from Betaproteobacteria class. 10-) RBH with all complete genomes from Gammaproteobacteria class. 11-) RBH with all other complete genomes.

Analysis of the core and accessory regions of GDI is important in order to understand its evolution and adaptation to the plant environment [18]. Even though Pal5 is the first *Gluconacetobacter diazotrophicus *strain to be sequenced, it is possible to identify the core genome by comparing with closely related species. The closest completed genomes available in the database were identified by phylogenetic analysis (Additional file 3). These include *Acidiphilium cryptum *JF-5 (ACC), *Gluconobacter oxydans *621H (GOX) and *Granulibacter bethesdensis *CGDNIH (GRB). Using quartops analysis (quartets of orthologous proteins [19]) we identified 894 CDS as core. Most of these CDS are related to metabolism, information transfer and energy metabolism, as illustrated in figure 1. As CDS with low GC3 (G+C content of synonymous third position) are potential accessory genes, the mean and standard deviation of the non-quartops were used as cut-offs to identify possible accessory genes. We found that 1,352 CDS have a GC3 percentage lower than 80% (figure 2). Interpolated Variable Order Motifs [20] (IVOMs) were used to complement the accessory genome analysis, revealing that 1,164 CDS have an "Alien score" greater than the threshold, 11,134. The group of CDS in common between GC3 and IVOMs (851 CDS) was used to define the accessory genes in the genome. The percentages of conserved hypothetical proteins, hypothetical proteins, phage/IS elements and pseudo genes are higher in the putative accessory regions than in the core regions and in the genome (figure 1), suggesting that the putative accessory regions have been transferred horizontally into the genome. Overall, the putative accessory regions cover approximately 24% of the GDI genome and are separated into 28 distinct regions, of which seven are classified as phage regions (Additional file 4). A third and completely independent method, PHX, also supports the assignment of the predicted accessory regions (figure 3).

### Genome Islands: Variation among *G. diazotrophicus *strains

Because HGT is an important source of intra-specific genetic variation in bacteria [21], we investigated whether there are differences in putative genome islands among 19 *G. diazotrophicus *strains and one *G. johannae *strain, using PCR with primers designed against 39 single-copy genes in 20 Genome Islands (GIs), and 17 CDS from the core genome. There was a complex variation among the strains, with gene content of eleven GIs - 1, 3, 7, 8, 9, 11, 15, 16, 17, 18, 19 - either almost entirely conserved or less than 50% variable (Additional file 5). In two GIs - 12 and 14 - there was high variability in a group of genes, while other genes were conserved in most strains. The remaining seven GIs, representing approximately 7% of the genome, were highly variable, especially GIs 4 and 21, which are 78 and 242 kb long, and encode 80 and 242 CDS, respectively. Furthermore, a considerable number of CDS in these two GIs encode genes involved in processes that could confer a competitive edge, such as oxidative stress, proteases, biosynthesis of antimicrobial agents, amino acid metabolism and secondary metabolites, as well a large number of transport systems and transcriptional regulators. Both GI4 and GI21 also contain complete copies of the T4SS operon. As it has been suggested that T4SS can increase host adaptability in *Bartonella *[22], we suspected that they could be a source of intraspecific variation among *G. diazotrophicus *strains. A Southern blot used to probe the *trbE *gene shows that indeed the T4SS copy number varies from one to four depending on the strain (Additional file 6). These GIs could be especially important for bacterial adaptation to the endophytic lifestyle and may confer adaptation advantages to *G. diazotrophicus *in comparison with other microbes that colonize the same niche.

### General Comparison

As the experimental results support the prediction of accessory regions in GDI, another interesting question concerns which regions of the genome resembles genomes from the database. For this purpose, a Reciprocal Best Hits (RBH) comparison was used [23]. The RBH analysis indicates that only 2,966 CDSs of GDI generate a hit with a completed bacterial genome. Among them, 2,470 CDSs have best hit with the Alphaproteobacteria class, 190 with the Betaproteobacteria class, 188 CDS with the Gammaproteobacteria class and 118 with other groups. The distribution of all RBHs demonstrated that even genes from phylogenetically distant related organisms can exhibit high percent identity (Additional file 7). The organism with the highest number of best hits is GOX, with 1,099. However, in figure 1, it is possible to observe that most of the hits occur in core regions. In the three organisms closest to GDI, around 90% of the best hits occur in core regions, with 10% in accessories regions. On the other hand, among rhizobiales and other Alphaproteobacteria orders, 56% of the best hits occur in core regions and 44% in accessory regions (Additional file 8). Curiously, complete genomes from the Betaproteobacteria class, Gammaproteobacteria class and other groups have a significant number (65-70%) of RBHs in core regions, and low percentage (30-35%) in accessory regions. In addition, the number of RBHs with phytopathogenic organisms is higher in Betaproteobacteria and Gammaproteobacteria than in Alphaproteobacteria (68%, 55% and 8%, respectively).

### Comparisons with other endophytic bacteria

Currently, there are only nine complete genome sequences of endophytic bacteria, and all are Proteobacteria. Using the complete genomes, we searched for common and exclusive CDS among endophytic bacteria in order to identify genes that could explain the endophytic capacity. However, we found only five CDS that are exclusively conserved (Additional file 9). The comparison among the endophytic organisms indicates that GDI has more CDS exclusively conserved with *Methylobacterium populi *BJ001 (133 genes) than with the others, which is consistent with the fact that *M. populi *BJ001 is also an Alphaproteobacteria. Most of these genes (Additional file 10) occur in an accessory region (GI4, GI9, GI12, GDI13, GDI14, GDI19 and GI21), and many are putative transcriptional regulators and putative T4SS (Additional file 9), which could also be involved in bacteria-host interactions. We also searched for exclusively conserved CDS between GDI and *Azoarcus sp*. BH72, as these two bacteria are currently the only diazotrophs among the endophytes sequenced. The result confirmed the presence in both endophytes of the *nif *cluster (figure 2, around 0.5 MB) and genes from the putative *gum *cluster are only conserved within *Azoarcus sp*. BH72 and GDI (Additional file 10). An assessment of the classes and frequency of signaling CDS in both diazotrophs shows that *Azoarcus sp*. BH72 has acquired a far more complex set of regulators (Additional file 11). In contrast, GDI has many more transport systems than *Azoarcus sp*. BH72 (Additional file 12). Altogether, the strategy developed by GDI to interact with plants seems to be more similar to *Methylobacterium populi *BJ001 then to other endophytes. However, the result suggests that there is not only one strategy and probably there are different ways in which bacteria can interact with plants.

After we completed this work, a second genome sequence of *Gluconacetobacter diazotrophicus *strain Pal5 was deposited. We carried out extensive comparisons between the two sequences. The comparison is summarized in Additional file 13. The results show significant differences between the two versions. GDI-BR has 309 more CDS than GDI-US, although this number is significantly reduced when small ORFs are annotated as CDSs in GDI-US. Likewise, the number of unique genes in both genomes decreases from 747 and 438 to 624 and 110, respectively, when the small CDSs are taken into account. The results show that the transposases, integrases and hypothetical proteins can explain the majority of the differences between the two sequences. Furthermore, 67% of the genes unique to GDI-BR are located in Genome Islands. On the other hand, 85% BBH among the two sequences are found outside the GIs. The results of the genomic comparisons between the two sequences are compatible with the PCR results reported here, that showed that most of the genic differences among GDI strains are situated in the GIs. Furthermore, when GIs from the two sequences are compared, most of the genic variation is found in the same more variable GIs (data not shown). Altogether, these analyzes suggest that the two sequences deposited as *G. diazotrophicus *Pal5 strain may represent either two different strains or a fast diverging strain.

In addition, our results were corroborated by at least three independent approaches. First, Southern Blot analyzes confirmed that the genomic sequence we have deposited has 4 copies of the TSS4 secretion system. Furthermore, PCR with primers that amplified genes in the GIs verified the presence of all CDS in our sequence, while some like GDI2782 which encodes a putative H(+)/Cl(-) exchange transporter, is absent from the second sequence. Finally, over 500 CDS in our sequence were validated by proteomics [17]. Some of these CDS may confer unique biological properties and competitiveness to *Gluconacetobacter diazotrophicus *Pal5, such as a Bacteriocin (GDI0415). Additional file 14 contains the list of Blast Best Hits among the two *Gluconacetobacter diazotrophicus *Pal5 genomic sequences, a list of unique CDS found in chromosome from GeneBank file CP001189 and a list of unique genes found in chromosome from GeneBank file AM889285 (this work).

### Genome Features in Core Regions

#### Osmotolerance

**Figure 4:**
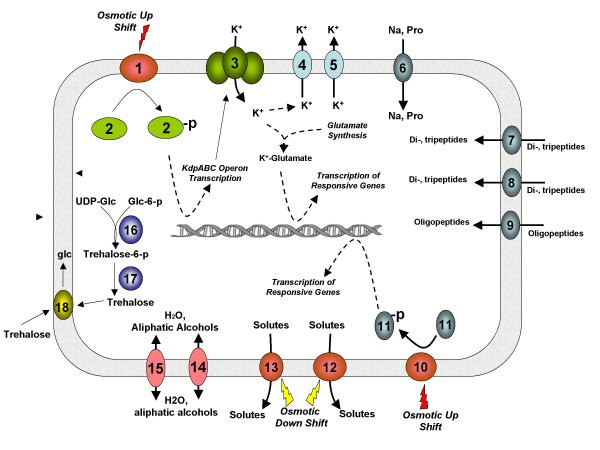
**Osmotolerance mechanisms in *G. diazotrophicus***. Osmotolerance mechanisms in *G. diazotrophicus*. (1) Sensor protein *kdpD *(GDI1564). (2) Transcriptional regulatory protein *kdpE *(GDI1565). (3) Potassium ABC transporter (*kdpABC *transporter; GDI1566-1568). (4) Glutathione-regulated system protein *kefB *(GDI0899) and (5) *kefC *(GDI2585). (6) Proline/betaine transporter (GDI2530). (7) Dpp ABC transporters for di- and tripeptides (GDI0246-GDI0250, GDI0454-GDI0458 and GDI3540-GDI3544). (8) Transporter *dtpT*, (GDI3819 and GDI0829). (9) Oligopeptide transporter (Opt; GDI3108). (10) Sensor kinase EnVZ (GDI3087). (11) OmpR (GDI3088). (12) Large Conductance MS channel *mscL *(GDI1732). (13) Small conductance MS channel, *mscS*, (GDI0793, GDI1149, GDI1789, and GDI3802). (14) *glpRDFK *(GDI1751-1754). (15) *glpDKF *(GDI0262, GDI0266, and GDI0267). (16) *otsA *GDI0917. (17) *otsB *GDI0916). (18) Periplasmic trehalase (*treA *GDI1341). The function of the proteins was verified by BLAST and motif searches of the corresponding CDS against public databases.

GDI supports high sugar concentrations, being able to tolerate up to 30% sucrose, but is sensitive to salt [24]. This shows its adaptation to sugarcane tissues, where the sucrose content is frequently high. Several osmoprotection systems were found (figure 4). There is a Kdp sensor system *kdpABCDE*, which regulates potassium flux (GDI1564-1568) [25]. One putative proline/betaine transporter gene was detected (GDI2530), but transporter genes *proU*, *betT *and *opuA *were not found. Pathways for glycine/betaine production are incomplete and genes necessary for conversion from choline to betaine are absent. The GDI genome harbors three Dpp ABC transporters that facilitate the uptake of di- and tripeptides (GDI0246-0250, GDI0454-0458 and GDI3540-3544). Two ORFs encoding a DtpT transporter, also involved in the uptake of di- and tripeptides, are present (GDI3819 and GDI0829). The presence of *otsA*, *otsB *and *treA *homologs (GDI0917, GDI0916 and GDI1341) suggests that GDI may synthesize and use the osmolytic disaccharide trehalose, although experiments on solid culture medium have shown that GDI is able to grow poorly on trehalose as a carbon source (data not shown). The hyperosmotic sensing in GDI may occur through the two-component system *envZ/ompR *(GDI3087 and GDI3088). However, the *envZ*-regulated porins *ompF *and *ompC *are not present. In bacteria, two porins (*aqpZ *and *glpF*) regulate the movement of water and aliphatic alcohols across cell membranes [26]. Homologs of *aqpZ *are missing in GDI, although two sets of glyceroporin genes were found in two clusters: one containing *glpRDFK *(GDI1751-1754) and the other composed of *glpDKF *(GDI0262, GDI0266, and GDI0267). The mechanisms shown in figure 4 and discussed here are similar to those found in bacteria without the high level of tolerance to high sugar concentrations observed for *G. diazotrophicus*. Therefore, unknown mechanisms that protect the bacteria specifically against high sugar concentrations may act in GDI. However, GDI seems to have a larger number of isoforms of enzymatic systems involved in osmotolerance. These differences may be explained by the different niches inhabited by GDI and *Azoarcus sp *BH72. While GDI is found in plants with elevated concentration of sugars, *Azoarcus sp *BH72 lives in association with plants that do not accumulate carbon sources in high concentration in vegetative tissues, rice and Kallar grass, and thus *Azoarcus sp *BH72 may not need a large number of enzymes.

### Acid tolerance

GDI has high tolerance to low pH and organic acids and is able to fix nitrogen at pH values as low as 2.5 [27]. The acidophile *Acetobacter aceti *has an unusual citric acid cycle (CAC) that is important for acetic acid resistance at low pH [28]. Genome analyses revealed the presence in the GDI genome of homologs of the alternative *A. aceti *citrate synthase gene *aarA *(GDI1830) and the gene for an acetyl-CoA hydrolase family protein gene with succinyl-CoA:acetate CoA-transferase activity, called *aarC *(GDI1836). In GDI, the *aarAC *homologs occur in a cluster similar to that of *A. aceti*, contrasting with the organization of these genes in non-acidophilic species, thus indicating that the same mechanisms of acid tolerance involving the CAC may be acting in both organisms. We also found a homolog of an ABC-transporter gene *aatA *(GDI1739) that, in *A. aceti*, constitutes an organic acid efflux pump mediating resistance to several acids [29]. An unusual observation is the presence in the GDI genome of two copies of the chaperonin genes *groES *(GDI2050, GDI2648) and *groEL *(GDI2049, GDI2647), which are usually present as single copy in bacteria. In *A. aceti*, overexpression of the *groESL *operon led to augmented resistance to acetic acid [30], which may be explained by the fact that chaperonins protect proteins under denaturing conditions such as low pH [31].

### Polysaccharides: CPS, EPS and LPS

Cell-surface components that are commonly involved in plant-bacteria interactions include capsular polysaccharides (CPS), exopolysaccharides (EPS), and lipopolysaccharide (LPS). On the GDI chromosome we found nine CDS related to polysaccharide encapsulation (GDI2398 to GDI2402 and GDI2409 to GDI2413). The GDI genome contains several CDS related to lipopolysaccharide biosynthesis. Five CDS (GDI3265, GDI1647, GDI1652, GDI1447 and GDI0495) encode glycosyltransferases, three CDS (GDI2535, GDI2549 and GDI2493) may be involved in lipopolysaccharide transport, one CDS (GDI2975) encodes an O-antigen polymerase, and there is an ADP-heptose synthase (GDI1133) and a nucleotidyl transferase (GDI0713). Seven CDS (GDI2490, GDI2971, GDI2492, GDI2544, GDI2549, GDI1898 and GDI1899) related to the synthesis of other EPS such as beta-glucans and exooligosaccharides were also identified. These CDS are dispersed over the GDI genome and encode *exoF*, *exoZ*, *exoY*, *exoO*, *exoP*, *exoN *and *exoC*, respectively. Homologs of these CDS are involved in the interaction between rhizobia and their host plants [32]. GDI has a cluster (GDI2535-GDI2552) containing 14 CDSs that is similar to the *gum *cluster of *Azoarcus sp*.BH72, *X. campestris *and *X. fastidiosa*. The *gum *cluster in *X. campestris *is responsible for the synthesis of an EPS that is involved in host plant colonization and virulence [33]. However, not all genes from the gum operon are present in GDI. We found eight CDSs (GDI2552, GDI2549, GDI2547, GDI2538, GDI2550, GDI2535, GDI2542 and GDI2548) which represent the genes *gumB, C*, *D*, *E*, *H*, *J*, *K and M*, respectively. The genes *gumF*, *G*, *I *and *L *are not present in the GDI genome. As GDI is not virulent, this cluster may be related with colonization and survival. In addition, it is proposed that the viscous nature of EPS helps localize and stabilize hydrolytic enzymes produced by the bacteria [34]. We found a putative endoglucanase protein (GDI2537) in the *gum *cluster that may degrade plant cell walls in order to facilitate the active penetration of the bacteria and thereafter the colonization. In addition, the production of hydrolytic enzymes by GDI has been observed [35].

### Biological Nitrogen Fixation (BNF)

The genetics and biochemistry of BNF and nitrogen utilization by *G. diazotrophicus *have been previously investigated to some extent. Corroborating previous studies [36], we have found that the GDI structural genes for nitrogenase *nifHDK *are arranged in a cluster (GDI0425-GDI0454), which also contains other N_2 _fixation-related genes, such as *fixABCX*, *modABC *and *nifAB*. Other related genes, *ntrX*, *ntrY *and *ntrC *(GDI2263, GDI2264, and GDI2265) are localized elsewhere in the chromosome in a 5.2 kb cluster. There are three copies of *nifU *homologous genes, one localized in the *nif *cluster (GD0447), and the other two scattered on the GDI chromosome (GDI1392 and GDI3055). No *draT *or *draG *homologs were found in GDI, confirming that nitrogenase activity is not regulated at the post-translational level. It has been suggested that post-translational modulation in *G. diazotrophicus *might be mediated by a FeSII Shethna protein [37], but no such CDS was identified. However, many other FeSII protein genes are present, and they possible candidates for this role. The apparent absence of *nifL *as a *nifA *activity modulator in response to the cell O_2 _status in GDI [38] is in agreement with the lack of a *nifL *homolog on the genome. The *nifA *protein appears to be inherently sensitive to O_2_. In *G. diazotrophicus*, the main route for assimilation of ammonia is believed to occur through the glutamine synthetase/glutamate synthase pathway (GS/GOGAT encoded by *glnA *and *gltDB*, respectively) [39]. However, the genome analysis suggests the existence of alternative routes, where the putative enzymes NAD-synthase (GDI0919), aminomethyltransferase (GDI2317), histidine ammonia-lyase (GDI0550) and D-amino acid dehydrogenase (GDI2422) would incorporate ammonia into different compounds. The enzymatic activity of GS is known to be regulated by an adenylyltransferase enzyme, which is probably encoded by *glnE *(GDI3425). The glutamate dehydrogenase gene was not found in GDI, although its activity was demonstrated for *G. diazotrophicus *strain Pal3 [38].

### Signaling and quorum sensing

The GDI genome contains 16 GGDEF family genes that are involved in the synthesis of the second messenger cyclic di-GMP, which has been shown to regulate cellulose synthesis and other processes such as transitions between sessile and planktonic lifestyle and pathogenesis [39]. There are three cytoplasmic and 14 membrane-bound histidine kinase signaling proteins, the majority of which form two-component signaling systems with a neighboring response regulator gene. Among these histidine kinases are homologs of the *kdpD *(GDI1566), *envZ *(GDI3079), *chvG *(GDI1265), *ntrY *(GDI2264), *ntrB *(GDI2266) and *phoB *(GDI3817) genes. In addition, there are two adjacent hybrid histidine kinase/response regulator genes that are organized in an apparent operon (GDI3283-3293) that contains several chemotaxis genes and a proteolytic system encoded by *hslUV *that is absent in GOX. Chemotaxis enables microorganisms to move towards beneficial or away from harmful substances in their environments by means of flagellar motility. The *G. diazotrophicus *genome contains nine methyl-accepting proteins (MCPs, chemotaxis sensor proteins), the majority of which have close homologs in rhizobia, but not in the phylogenetically related non-endophyte GOX, which has only three MCP genes [40]. Quorum sensing has been shown to be important in traits such as virulence, biofilm formation and swarming motility in many bacteria [41]. In the *Azoarcus sp *BH72 genome, quorum sensing genes were not found, and it was suggested that this was compatible with a non-pathogenic interaction of *Azoarcus sp *BH72 with the host plant [12]. Nevertheless, GDI, which inhabits a niche similar to *Azoarcus sp *BH72, has three quorum sensing genes: one *luxI *autoinducer synthase gene (GDI2836) and two *luxR*-type transcriptional regulator genes (GDI2837, GDI2838). Quorum sensing genes are also present in several rhizobial genomes, and they play roles in nodulation and nitrogen fixation [42].

### Plant Growth-Promoting (PGP) Traits

There are several indications that GDI promotes plant growth by more than a few independent mechanisms besides nitrogen fixation, including synthesis of phytohormones and increased uptake of nutrients [43]. Recent work has shown that mutations in two genes involved in cytochrome c biogenesis reduced auxin levels to 10% of the wild-type strain [44], suggesting their involvement in indole acetic acid (IAA) production, and indicating that GDI has at least two independent pathways for auxin biosynthesis. In addition, characterization of the IAA biosynthetic route in GDI has shown that auxin is mostly synthesized by the Indole-3-pyruvic acid (IPyA) pathway [44]. Although no CDS encoding an indole 3-pyruvate carboxylase was found in GDI genome, we cannot rule out that the biochemical activity could be executed by one of the many putative decarboxylases identified in the genome. The presence of genes encoding enzymes such as aromatic-L-amino-acid decarboxylase (GDI1891), amine oxidase (GDI1716) and aldehyde dehydrogenases (GDI0311, GDI0461, GDI640) suggests that the bacteria might synthesize IAA via the trypamide pathway (TAM). Also, the presence of two genes coding for putative nitrilases (GDI0018, GDI3743) suggests that IAA might be produced by the indole-3-acetonitrile pathway (IAN). In addition to phytohormone production, some rhizosphere-associated bacteria can stimulate plant growth by secreting a mixture of plant volatiles, mainly 3-hydroxy-2-butanone (acetoin) and 2,3-butanediol [45]. Although the role of GDI in PGP has been studied, no attention has been paid to the production of volatiles. We found GDI is likely to be capable to synthesize acetoin once the genome sequence encodes two enzymes of the pathway; acetolactate synthase (GDI0022, GDI0023) and acetoin diacetyl reductase (GDI2623). In addition, although an acetolactate decarboxylase has not been identified, 2-acetolactate can be converted to diacetyl spontaneously in the presence of oxygen (46). It has been shown for *Azospirillum brasilense *that the production and secretion of polyamines promote plant growth [47]. The presence of genes coding for enzymes for the synthesis (GDI0476, GDI2322) and secretion (GDI2595) of spermidine in the *G diazotrophicus *Pal5 genome sequence suggests that this polyamine may also contribute to PGP. *G. diazotrophicus *has been shown to synthesize the gibberellins GA1 and GA3 [48]. Although the gibberellin biosynthesis machinery in bacteria is largely unknown, recent studies have suggested likely biosynthetic mechanisms in *Bradyrhizobium japonicum *[49]. The GDI genome contains genes for the synthesis of the diterpenoid precursor isopentenyl diphosphate through the non-mevalonate pathway. Condensation reactions of this precursor to form geranylgeranyl diphosphate may be performed by the geranyltranstransferase *ispA *(GDI1861). However, homologs of the genes responsible for the cyclization of geranylgeranyl diphosphate in *B. japonicum *(ent-copalyl diphosphate and ent-kaurene synthase) are apparently absent in the GDI genome and therefore the mechanism of cyclization of geranylgeranyl diphosphate to ent-kaurene remains unknown. However, a putative squalene cyclase (GDI1620) could fulfill such function, since a study with recombinant squalene cyclase has shown some cyclization of geranylgeraniol by this enzyme [50]. Oxidation steps of ent-kaurene, necessary to produce GA_1 _and GA_3_, may be catalyzed by two cytochromes P450 (GDI2364 and GDI2593), homologs of which are absent in other acetobacteraceae genomes, thus suggesting a likely specific role in *G. diazotrophicus*. It has been reported that the capacity of *G. diazotrophicus *to antagonize diverse plant pathogens such as fungi, and bacteria contributes to increasing its ability to survive under environmental stress and leads to an improvement in plant fitness which may have important consequences for agricultural productivity [9,10]. Its genome sequence encodes a large repertoire of genes whose products oppose attack from competing microbes, such as drug efflux systems, and acriflavin and fusaric acid resistance proteins. On the other hand, GDI may also produce a broad variety of proteins such as lytic enzymes and phospholipases and antibiotic biosynthetic pathways that could be toxic to other organisms. The secretion of a lysozyme-like bacteriocin by *G. diazotrophicus*, for instance, inhibits *Xanthomonas albilineans *growth [9]. Indeed, GDI encodes a putative lysozyme-like bacteriocin (GDI0416 and GDI0415).

### Sugar metabolism and energy generation

Sucrose is the common carbon source used for isolation of *Gluconacetobacter diazotrophicus *from sugarcane and other plants in the semi-solid LGIP medium [51]. However, sucrose is not directly metabolized by the bacteria. Experimental evidence has shown that there is a constitutively expressed levansucrase (LsdA GDI0471), secreted to the periplasm via a specific signal peptide-dependent pathway, that converts sucrose to beta-1,2 -oligofructans and levan [52]. In addition, a fructose-releasing exo-levanase (LsdB GDI 0477) probably controlled by an antitermination inducer system converts polyfructans into fructose [53]. A type II secretion operon (GDI481-GDI 490) is required for the transport o f LsdA across the outer membrane [54]. The transport of LsdB to the periplasm involves the cleavage of the N-terminal peptide signal, and it is induced during growth of the bacteria with low fructose levels but repressed by glucose [55].

In *G. diazotrophicus *oxidation of glucose to gluconate in the periplasmic space is the first step in glucose metabolism by GDI [56]. Gluconate may be synthesized by the product of three CDS encoding membrane-bound quinoprotein glucose dehydrogenases (GDI3277, GDI0325 and GDI0539) in accordance with the observed high activity of PQQ-GDH detected in glucose-containing batch culture of GDI strain Pal3 grown mainly under biological nitrogen fixation and/or C-limitation conditions [57]. A NAD-GDH (GDI2625) also participates in the glucose oxidation (intracellularly) when glucose is in excess [57]. Further periplasmic oxidation of gluconate to 2-ketogluconic acid occurs by a putative three-subunit flavin-dependent gluconate-2-dehydrogenase (GDI0854, GDI0855 and GDI0856). Gluconate dehydrogenases (extracellular, dye-linked and intracellular, NAD-Linked) activities have been demonstrated in GDI strain Pal3 grown in presence of gluconate with 2-ketogluconate the major compound accumulated (57). The production of 5-ketogluconate and 2,5 di-ketogluconate are probably mediated by a glucose/methanol/choline oxidoreductase (GDI0859) and a putative alcohol dehydrogenase cytochrome c/gluconate 2-dehydrogenase acceptor (GDI0860). High activities of 2-ketogluconate reductase (NAD linked) have been detected in a GDI Pal3 strain grown with gluconate [58].

CDS for transport (GDI3258) and phosphorylation (GDI0293) proteins indicate that gluconate can also be directly driven into the pentose phosphate route (PPP), supporting the experimental data [58]. The presence of a kinase (GDI3115), a 2-ketogluconate reductase (GDI 3432) and a 6-phosphogluconate dehydrogenase-NAD (GDI2166) corroborates with the experimental data which shows that the PPP is the main C-metabolism route in GDI following the oxidation of glucose to gluconate [57].

Different from GOX, CDSs encoding a complete respiratory chain complex I (*nuoA *- *nuoN or *complex I proton-translocating NADH-quinone oxidoreductase; GDI2459-GDI2471) are present in the GDI genome [59]. The GDI genome contains CDS that encode L-sorbosone dehydrogenases (GDI0574 and GDI3764), membrane-bound small and large subunits (GDI3280 and GDI3281) and the cytochrome c subunit (GDI3279) of aldehyde dehydrogenase, indicating that GDI may be able to synthesize the industrially important substances such as L-ascorbic acid (vitamin C) and its precursor 2-keto-L-gulonic acid [60].

### Genome Features in Accessory Regions

#### Type IV secretion system

Type IV secretion systems (T4SS) are multi-subunit cell envelope-spanning structures, ancestrally related to bacterial conjugation machines, that transfer proteins, DNA and nucleoprotein complexes across membranes [61]. Moreover, T4SSs have been described as essential pathogenicity factors and recently it has been indicated that TSS4 can also increase host adaptability in *Bartonella sp*. [22]. GDI has 4 complete T4SS in the chromosome which are similar to bacterial conjugation machines (*trb*) of *Agrobacterium tumefasciens *[62] and Ti (tumor inducing) *Enterobacter *IncP plasmid R751 [63]. Although the order of the *trb *genes in the operon is conserved (*trbB*, -*C*, -*D*, -*E*, -*J*, -*L*, -*F*, -*G*, -*I*), two genes are missing from the original *trb *operon (*trbK *and *trbH*). The gene *trbK *has been reported as non-essential but *trbH *has been reported as essential for conjugal transfer of *Agrobacterium *tumor inducing plasmid pTiC58 [63]. Another difference is that, in *Agrobacterium tumefasciens *and *Enterobacter *IncP plasmid R751, the first gene in the operon is *traI*, which is an essential signal for the quorum-sensing regulation of the Ti plasmid conjugation transfer [64]. In GDI the first gene in the operon is *traG*, which is essential for DNA transfer in bacterial conjugation. This gene is thought to mediate interactions between the DNA-processing (Dtr) and the mating pair-formation (Mpf) systems [65]. T4SS have been found in many different organisms [66], from pathogenic to mutualistic endosymbiont organisms (for instance, *Helicobacter pylori*, *Legionella pneumophila*, *Brucella spp*, *Bartonella spp*, *Rickettsia spp*., *Coxiella spp*., *Anaplasma marginale*, *Ehrlichia spp*, *Agrobacterium tumefaciens*, *Wolbachia spp*). All four complete T4SS operons in the GDI chromosome were found in accessory regions (GI4, GI12, and twice in GI21), suggesting that the bacteria acquired the ability to translocate macromolecules across the cell envelope to the plant. The four copies of the T4SS operon diverge by the presence of a variable region between the *traG *and the *trbB *genes that include transcriptional regulators *mucR *and *araC*, a DNA-binding protein HU-beta, an aldo/keto reductase and hypothetical proteins. These genes might confer specific functions to each T4SS copy.

### Flagella and pili

In many organisms, flagella are involved in motility, adherence, biofilm formation and host colonization [67]. GDI has a large accessory region (GI15) with at least 40 genes predicted to encode functions related to motility. This observation is in accordance with the presence of peritrichous flagella on the GDI cell surface. Next to the motility cluster there is a putative *tad *locus (*Flp-1*, *cpaABC*, *cpaEF*, *and tadBCDG*) which probably encodes the machinery for the synthesis of Flp (fimbrial low-molecular-weight protein) pili, which form a subfamily in the type IVb pilus family. In *Actinobacillus*, *Haemophilus*, *Pasteurella*, *Pseudomonas*, *Yersinia *and *Caulobacter*. Flp pili are essential for biofilm formation, colonization and pathogenesis [68]. Additionally, several pseudopilins (GDI0483, GDI0484, and GDI0485) were identified as part of a type II secretion system. Recently, it has been shown that flagella-less mutant of GDI was non-motile and displayed reduced capacity to form biofilm [69]. These findings suggest that these genes were acquired by HGT and play an important role in the interaction with the plant.

## Conclusion

Despite the potential impact of endophytes on the environment and on crop production, our current knowledge of their biology is limited. Analysis of the *G. diazotrophicus *PAL5 complete genome sequence provides important insights into the endophytic relationship, and suggests many interesting candidate genes for post-genomic experiments.

The genome reveals an unexpectedly high number of mobile elements for an endophytic bacterium; it is in fact the endophyte with the highest frequency of mobile genes per Mb of genome. The high number of mobile elements seems to be associated with a high number of HGT events. The analysis of HGT shows that most of the genes are more similar to genes from the order rhizobiales (40%), suggesting that a likely previous niche was located in the rhizosphere. Thus, a recent evolutionary bottleneck and consequent relaxation of selection, due to a possible change of niche, is probably the hypothesis that could best explain the high number of HGT [15].

In addition, to change niche from rhizosphere to endophytic, the bacteria should have features that would allow it to penetrate the plant. The putative *gum*-like cluster containing an endoglucanase could be important in this regard. Moreover, the limited similarity with the *gum*-like cluster from *X. campestris *and the absence of some genes found in *X. campestris *may mean that the cluster adapted to a non-virulence profile. However, the ability to penetrate the plant is not enough to transform it into an endophyte; the bacteria must evolve together with the plant to create a more depended relationship. The genome has many features to enhance plant fitness such as BNF, phytohormones and biocontrol genes, and all of them lie in the core of the genome or have a very low "Alien score". We propose that these features were important to create a dependent relationship, and may have helped GDI to spread out and occupy this niche. In contrast, many features that may be related to bacteria-plant interaction are found in genome islands, including type IV secretion systems, flagella, pili, chemotaxis, biofilm, capsular polysaccharide and some transport proteins. The overall result suggests that it is more likely that GDI acquired many features that are important for an endophytic lifestyle. Thus, experimental analyses of genes from genome islands may reveal an important source of gene candidates that will enhance our understanding of bacteria-plant relationship.

Finally, comparison of genome sequences of *Gluconacetobacter diazotrophicus *and *Azoarcus sp*. BH72 shows that these endophytic diazotrophic bacteria adopted very different strategies to colonize plants. A limited number of genomic features, such as the large number of TonB receptors, the *gum*-like and *nif *clusters, and osmotolerance mechanisms are common to both endophytic diazotrophic bacteria. On the other hand, *Gluconacetobacter diazotrophicus *has a larger number of transport systems, and it is capable of growing on a wide variety of carbon sources, while *Azoarcus sp*. BH72 has rather complex signaling mechanisms to communicate with its plant host.

## Methods

### Strain

*Gluconacetobacter diazotrophicus *strain PAl 5 (type strain) was isolated from sugarcane roots collected in Alagoas Sate, Brazil using the nitrogen-free semi-solid LGIP medium [2]. It was deposited at the Embrapa Agrobiologia Culture Collection and received the identification number BR 11281 (BR-stands for the Brazilian Nitrogen-fixing bacteria Culture Collection). Later on, this strain was deposited by Johanna Dobereiner at the American Type Culture Collection (ATCC 49037) and also at the Culture Collection Laboratorium von Microbiologie, Belgium (LMG 7603) [70].

### Genome sequencing, assembly and annotation

All the libraries were prepared with total bulk DNA originated from a Pal 5 lyophilized tube culture provided by the Embrapa Agrobiologia Culture Collection. Pal5 was grown in 500 mL Erlenmeyer flasks containing 200 mL of DYGS medium (Rodrigues-Neto et al., 1986) during 48 h at 200 rpm and 30°C. DNA extraction was performed according with the CTAB method [71]. Phenol: chloroform: iso-amilic alcohol (25:24:1) and chloroform: iso-amilic alcohol (24:1) washing steps were repeated 2 times to guarantee removal of cells debris and other contaminants during DNA extraction.

DNA shotgun libraries with insert sizes of 0.5-1 kb, 2-3 kb and 4-6 kb were constructed in pUC18 vectors and 10-17 kb in the cosmid pLARF3. Plasmid clones were end-sequenced on ABI377 and ABI3100 (Applied Biosystems) and MegaBACE 1000 (GE Healthcare) sequencers. A total of 103,506 high-quality reads were obtained and assembled into contigs using the Phrap assembly tool. For gap closure, 16,963 additional reads were obtained through PCR direct sequencing and primer-walking on plasmids. Manual editing was done using the GAP4 software package [72]. Genome integrity was verified by a physical map constructed using PFGE and hybridization with 42 single-copy and rDNA probes [73]. Initial automatic gene prediction was done using GLIMMER [74], and subsequently manually curated with reference to codon-specific positional base preferences. Before the manual annotation of each predicted gene, different tools were used. Similarity search was performed against different databases including Uniprot [75], PROSITE [76], nr, Pfam [77], and InterPro [78]. Additionally, SignalP [79], TMHMM [80] and tRNAscan-SE [81] were applied. All the data were viewed within the Artemis [82] program where the function of each gene was manually curated.

### Annotation colors

Pathogenicity/Adaptation/Chaperones, dark blue; Energy metabolism (glycolysis, electron transport etc.), gray; Information transfer (transcription/translation, DNA/RNA modification), red; Surface structures (IM, OM, secreted, LPS)), green; Stable RNA, cyan; Degradation of large molecules, light blue; Degradation of small molecules, purple; Central/intermediary/miscellaneous metabolism, yellow; Unknown and conserved hypothetical, orange; Regulators, magenta; Pseudogenes and partial genes, black; Phage/IS elements, pink; miscellaneous information (e.g. Prosite but no function), brown.

### Nucleotide sequence accession numbers

The genomic sequence reported in this article has been deposited in the EMBL database under accession numbers AM889285, AM889286 and AM889287. The genome annotation and features are available at .

### Core and accessory regions

The core regions were determined by quartops analysis (quartets of orthologous proteins), using reciprocal best hit of Blastp. The accessory regions were determined by a combination of two different methods: GC3 and IVOMs. The GC3 analyzes the percent of GC in the third base of the codon in each gene. For both methods, the regions indicated as accessory genes were manually checked for integrases, tRNAs and repeats (direct and inverted). The beginning and end of each the accessory region were defined by both methods and, in the case of bacteriophages, the genome islands were extended when evidence of the insertion point was found.

### Reciprocal Best Hits

Reciprocal best hits comparison was done using only the complete bacterial genomes publicly available at . Only reciprocal best hits with identity greater of 30% and alignment greater than 70% were selected.

### Plant Endophyte comparison

Six complete endophyte genomes were used to represent the endophyte group and three closest complete genomes phylogenetically to GDI were used to represent the core genome. Endophyte genomes were *Azoarcus sp*. BH72, *Burkholderia phytofirmans *PsJN, *Enterobacter sp*. 638, *Methylobacterium populi *BJ001, *Pseudomonas putida *W619 and *Serratia proteamaculans *568. Core genome species were *Acidiphilium cryptum *JF-5, *Gluconobacter oxydans *621H and *Granulibacter bethesdensis *CGDNIH. Only reciprocal best hits with more than 30% identity and 70% alignment were accepted.

## Abbreviations

BNF: Biological Nitrogen Fixation; GDI: *Gluconacetobacter diazotrophicus *PAL5; HGT: Horizontal Gene Transfer; GI: Genome Island; CDS: Coding Sequences; PHX: Predicted Highly Expressed Genes; T4SS: Type IV secretion system; ACC: *Acidiphilium cryptum *JF-5; GOX: *Gluconobacter oxydans *621H; GRB: *Granulibacter bethesdensis *CGDNIH; GC3: G+C content of synonymous third position; IVOMs: Interpolated Variable Order Motifs; IS: Insertion sequence; BBH: Blast Best Hit; Flp: fimbrial low-molecular-weight protein; bp: base pairs; Dtr: DNA-processing; Mpf: mating pair formation; RBH: Reciprocal Best Hits.

## Authors' contributions

PF coordinated the study.  MB, AP, DC, TU, WK, PB, LP, DF, HM, JJ, VF, BF, AB, ES, GA, AN, RA, DC, DO, TS, JM, AV, PG, EN, VP, JA, AO, LR, HG, ER, JB, LF, VM, RS, ML and PF conducted genome sequencing. MB and ML conducted genome assembly. MB, LL, PB, SR, GF, VF, MF, GM, AC, AN, RA, JM, HG, EN, VP, ML, LS, JM, KT, JA, MV, SS, AO, LR, JB, CM, AH, SA, AMC, WA, MD, RS, EO, AR, ML, OM and PF performed sequence annotation.  MB performed bioinformatics analyses and comparative genome analyzes. MB, SS, VP, EN, AO, LR, JB, CR, AMC, and PF analyzed the results and participated in writing sections of the manuscript. MB and PF assembled and wrote the final version of the manuscript. All authors read and approved the final manuscript.

## Supplementary Material

Additional file 1 **Distribution of mobile elements in plant endophyte complete genomes**. The percentage column: Percentage of total number of mobile elements from all CDS annotated on the endophyte complete genomes. Click here for file

Additional file 2 **Predicted Highly Expressed (PHX) genes**. The PHX and proteomic analysis was used to indicate potentially important genes in the GDI genome. Click here for file

Additional file 3 **16S phylogenetic tree from Alphaproteobacteria**. The Neighbor joining phylogenetic tree of 16S from Alphaproteobacteria was done using ClustalX. In blue are the three completed genomes closest to *G. diazotrophicus *PAL5 available in GenBank. Click here for file

Additional file 4 **The 28 genome islands (GI) identified by GC3 and IVOMs**. The GI column has the ID for each genome island. The integrase column shows which kind of integrase was found in each genome island. The CDS column shows how many CDS are inside the genome island. The Alien+GC3 column show how many CDS in each genome island were identified as accessory by both methods. The Related column shows which kinds of genes were found in each genome island. Click here for file

Additional file 5 **Variation in *G. diazotrophicus *strains**. 20 different strains were tested for gene variation. 37 CDS were selected from 21 putative genome islands and 17 CDS were selected from putative core regions of the chromossome as control. (+): PCR positive. (-): PCR negative. Click here for file

Additional file 6 **Presence of homologues of the *trbE *gene among *G. diazotrophicus *strains**. Total DNA of 11 *Gluconacetobacter *strains was completely digested with restriction enzymes EcoRI (a.) or EcoRV (b.), separated on agarose gel and submitted to Southern blot analysis using a fragment of CDS GDI0133 (*trbE*, part of type IV secretion system) as a probe. Numbers 1-10 represent *G. diazotrophicus *strains: Pal5 (1), 3R2 (2), URU (3), 38f2 (4), PRJ50(5), Pal3 (6), AF3 (7), PCRI (8), PPe4 (9), CNFe-550 (10). Number 11 represents *G. johannae*. In strain Pal5, only 3 bands are present, although the genome sequence indicates the presence of four copies of the trbE gene. However, the fourth *trbE *paralog (GDI1016) is more dissimilar to the probe sequence then the other three (GDI0133, GDI2742 e GDI2911), which may have prevented hybridization. Click here for file

Additional file 7 **Distribution of percent ID from RBH results**. In red all the RBH from Rhodospirillales order. In yellow all RBH from other Alphaproteobacteria class and in blue RBH from other genomes beside Alphaproteobacteria class. Click here for file

Additional file 8 **Number of Reciprocal Best Hits (RBH) in accessory and core regions**. The first column shows the number of RBH for each organism in parentheses. The RBH in columns show the total number of RBH for each organism. The RBH % by organism columns shows the percent of RBH in relation with the total number of RBH found in accessory and core regions. RBH result has 708 RBH in accessory regions and 2,258 in core regions. The RBH % by organism columns shows the percentage of RBH in accessory and core regions for each organism or group. GOX = *Gluconobacter oxydans *621H, GBE = *Granulibacter bethesdensis *CGDNIH, ACR = *Acidiphilium cryptum *JF-5, Rhiz = All the complete genomes from Rhizobiales order, Other Alpha = All other complete genomes from Alphaproteobacteria class, Beta = All complete genomes from Betaproteobacteria class, Gamma = All complete genomes from Gammaproteobacteria class, Others = All other complete genomes. Click here for file

Additional file 9 **Endophyte comparison gene list**. Endophyte gene list. Click here for file

Additional file 10 **Endophyte comparison**. In gray, genes similar to all genomes (core + endophyte, see Methods). In blue, genes present in all endophyte but not in core genomes. In red, genes only similar to GDI and *Azoarcus sp *BH72. In purple, genes only similar to GDI and Methylobacterium populi BJ001 and in green genes that are only present in GDI and at least two other endophyte genome. Click here for file

Additional file 11 **Comparison of main signalling protein categories**. AT, *Agrobacterium tumefaciens *C58; BJ, *Bradyrhizobium japonicum *USDA110; ML, *Mesorhizobium loti *MAFF303099, SM, *Sinorhizobium meliloti *1021, GO, *Gluconobacter oxydans *621H; RP, *Rickettsia prowazekii *MadridE; AB, *Azoarcus sp*. BH72; AE, *Azoarcus sp*. EbN1; XF, *Xylella fastidiosa *9a5c; EC, *Escherichia coli *K12-MG1655. Click here for file

Additional file 12 **Comparison of main transport-related protein categories**. AT, *Agrobacterium tumefaciens *C58; BJ, *Bradyrhizobium japonicum *USDA110; ML, *Mesorhizobium loti *MAFF303099, SM, *Sinorhizobium meliloti *1021, GO, *Gluconobacter oxydans *621H; RP, *Rickettsia prowazekii *MadridE; AB, *Azoarcus sp*. BH72; AE, *Azoarcus sp*. BH72, XF, *Xylella fastidiosa *9a5c; EC, *Escherichia coli *K12-MG1655. Click here for file

Additional file 13 **Comparison among the two *Gluconacetobacter diazotrophicus *Pal5 genomic sequences**. GDI-BR, NCBI RefSeq NC_010125, GDI-US, NCBI RefSeq NC_011365, GIs, Genome Islands. Click here for file

Additional file 14 **CDS list of the two *Gluconacetobacter diazotrophicus *Pal5 sequences**. Sheet 1: Blast best hits list of CDS found in both genomes. Sheet 2: List of unique CDS found in chromosome from GeneBank file CP001189. Sheet 3: List of unique genes found in chromosome from GeneBank file AM889285 (this work). Click here for file
