# Supplementary material for: Complete genome sequence of the sugarcane nitrogen-fixing endophyte Gluconacetobacter diazotrophicus Pal5
Source: BMC Genomics. 2009 Sep 23;10:450. doi: 10.1186/1471-2164-10-450 (PMC2765452; doi:10.1186/1471-2164-10-450)
Supplement: Additional file 1 — Distribution of mobile elements in plant endophyte complete genomes. The percentage column: Percentage of total number of mobile elements from all CDS annotated on the endophyte complete genomes. [file 1471-2164-10-450-S1.PDF]

Genomes	Integrases	Transposases	Total	CDS	Percent
Azoarcus sp. BH72	9	13	22	3,989	0,55%
Pseudomonas putida W619	18	16	34	5,182	0,66%
Enterobacter sp. 638	22	6	28	4,240	0,66%
Serratia proteamaculans 568	17	25	42	4,942	0,85%
Burkholderia phytofirmans PsJN	41	43	84	7,241	1,16%
Methylobacterium populi BJ001	19	65	84	5,365	1,6%
Gluconacetobacter diazotrophicus PAL5	55	190	245	3,930	6,23%
