# Supplementary material for: Complete genome sequence of the sugarcane nitrogen-fixing endophyte Gluconacetobacter diazotrophicus Pal5
Source: BMC Genomics. 2009 Sep 23;10:450. doi: 10.1186/1471-2164-10-450 (PMC2765452; doi:10.1186/1471-2164-10-450)
Supplement: Additional file 4 — The 28 genome islands (GI) identified by GC3 and IVOMs. The GI column has the ID for each genome island. The integrase column shows which kind of integrase was found in each genome island. The CDS column shows how many CDS are inside the genome island. The Alien+GC3 column show how many CDS in each genome island were identified as accessory by both methods. The Related column shows which kinds of genes were found in each genome island. [file 1471-2164-10-450-S4.PDF]

GI	Integrase	tRNA	Start	Stop	Size (bp)	CDS	IVOM+GC3	IVOM score	Relared
1	no	no	3,936,650	12,762	20,275	26	25	19	Conserved and hypothetical proteins.
2	no	no	25,516	33,049	7,533	6	6	26	Pseudo and transposase.
3	no	no	62,937	68,001	5,064	8	8	19	Conserved hypothetical.
4	Recombinase	Met	86,651	165,382	78,731	80	61	25	T4SS.
5	no	no	203,709	210,069	6,360	6	6	18	TonB and LysR.
6	Integrase	no	235,635	240,903	5,266	6	6	18	LysR and Na(+)/H(+) antiporter.
7	no	no	307,302	315,106	7,804	11	9	21	GGDEF family.
8	no	no	342,449	347,663	5,184	7	7	25	Arsenical pump.
9	Integrase	no	380,519	413,160	32,641	49	46	19	Lytic transglycosylase and Bacteriocin.
10	no	no	625,868	632,087	6,219	7	6	14	AsnC family.
11	Integrase	no	767,614	778,664	11,050	12	11	24	Deoxyribodipyrimidine phto-lyase.
12	Recombinase	Glu	968,093	1,044,357	76,264	80	69	29	Type IV and LysR family.
13	Integrase	no	1,058,198	1,117,943	59,745	87	79	27	Toxin anti-toxin system and regulators.
14	Phage integrase	Leu	1,597,584	1,541,820	55,764	63	59	33	Potassium transporting and TrwC.
15	no	Met	1,664,412	1,742,518	78,106	77	54	15	Flagellar and cheotaxis proteins.
16	Phage integrase	Leu	2,233,424	2,169,306	64,118	70	6	20	Organic solvent tolerance and outer membrane.
17	Phage integrase	Glu	2,309,161	2,298,385	10,776	19	19	33	Mobilization (plasmid transfer).
18	Phage integrase	Lys	2,392,259	2,428,554	36,295	31	2	17	Chemoreceptors and rod shape-determining.
19	Integrase	no	2,487,556	2,513,854	26,296	26	22	64	Capsule polysaccharide export.
20	Integrase	no	2,691,273	2,704,152	12,879	16	12	18	Conserved hypothetical proteins.
21	Recombinase	Met	2,776,876	3,007,766	230,890	242	191	28	NPRS, T4SS (2x) and LysR family.
22	no	Val	3,073,793	3,098,303	24,510	27	24	29	Fatty acids biosynthesis.
23	Phage integrase	Arg	3,644,620	3,672,641	28,021	43	7	20	Exported and membrane proteins.
24	Phage integrase	Try	3,672,862	3,695,512	22,650	33	24	20	Hypothetical proteins.
25	no	no	3,703,039	3,709,055	6,016	8	8	20	Lyase.
26	Integrase	no	3,774,785	3,781,006	6,221	7	7	31	Hypothetical proteins.
27	no	no	3,859,995	3,867,737	7,742	8	8	35	mismatch repair proteins.
28	no	Thr	3,882,753	3,893,554	10,801	12	12	16	TonB protein.
