# Supplementary material for: Complete genome sequence of the sugarcane nitrogen-fixing endophyte Gluconacetobacter diazotrophicus Pal5
Source: BMC Genomics. 2009 Sep 23;10:450. doi: 10.1186/1471-2164-10-450 (PMC2765452; doi:10.1186/1471-2164-10-450)
Supplement: Additional file 5 — Variation in G. diazotrophicus strains. 20 different strains were tested for gene variation. 37 CDS were selected from 21 putative genome islands and 17 CDS were selected from putative core regions of the chromossome as control. (+): PCR positive. (-): PCR negative. [file 1471-2164-10-450-S5.PDF]

Alien Island	ORF	Strain																				Possible function
		3R2	38F2	AF2	AF3	AF43	AR20	AR55	C1	C3	CFNE550	CFN-cf55	PAL3	PAL5^T	PBD4	PRC1	PPE4	PRJ2	PRJ50	UAP-cf58	URU	
1	GDI 0004	(+)	(+)	(+)	(+)	(+)	(+)	(-)	(+)	(+)	(+)	(+)	(-)	(+)	(+)	(+)	(+)	(+)	(+)	(-)	(+)	Conserved hypothetical protein
2	GDI 0029	(+)	(+)	(+)	(+)	(+)	(+)	(-)	(-)	(+)	(-)	(+)	(+)	(+)	(+)	(-)	(-)	(+)	(-)	(+)	(+)	Putative transposase
3	GDI 0061	(+)	(+)	(+)	(+)	(+)	(+)	(+)	(+)	(+)	(+)	(+)	(-)	(+)	(+)	(+)	(+)	(+)	(+)	(+)	(+)	Conserved hypothetical protein
4	GDI 0079	(+)	(-)	(+)	(-)	(-)	(-)	(-)	(-)	(+)	(-)	(-)	(-)	(+)	(+)	(-)	(-)	(+)	(-)	(-)	(+)	Catalase
	GDI 0083	(+)	(+)	(+)	(-)	(-)	(-)	(-)	(-)	(-)	(-)	(-)	(-)	(+)	(+)	(-)	(+)	(+)	(-)	(-)	(-)	Putative flavin oxidoreductase
	GDI 0121	(-)	(-)	(-)	(-)	(-)	(-)	(-)	(-)	(-)	(-)	(-)	(-)	(+)	(-)	(-)	(-)	(-)	(-)	(-)	(-)	Putative metallophosphoesterase
	GDI 0142	(+)	(-)	(+)	(-)	(-)	(-)	(-)	(-)	(+)	(-)	(-)	(-)	(+)	(+)	(-)	(+)	(-)	(+)	(-)	(-)	Putative multidrug resistance protein
7	GDI 0299	(+)	(+)	(+)	(+)	(+)	(+)	(+)	(+)	(+)	(+)	(+)	(+)	(+)	(+)	(+)	(+)	(+)	(+)	(+)	(+)	Putative signalling protein, GGDEF family
8	GDI 0335	(+)	(+)	(+)	(+)	(+)	(+)	(+)	(+)	(+)	(+)	(+)	(+)	(+)	(+)	(+)	(+)	(+)	(+)	(+)	(+)	Putative arsenical pump membrane protein
9	GDI 0415	(+)	(+)	(+)	(+)	(+)	(+)	(+)	(+)	(+)	(+)	(+)	(+)	(+)	(+)	(+)	(+)	(+)	(+)	(+)	(+)	Bacteriocin protein
11	GDI 0751	(+)	(+)	(+)	(+)	(+)	(+)	(+)	(+)	(+)	(+)	(+)	(-)	(+)	(+)	(+)	(+)	(+)	(+)	(+)	(-)	Putative deoxyribodipyrimidine photo-lyase
12	GDI 0989	(-)	(-)	(-)	(-)	(+)	(-)	(-)	(-)	(-)	(-)	(-)	(-)	(+)	(+)	(-)	(-)	(-)	(-)	(+)	(-)	Putative 2-dehydropantoate 2-reductase
	GDI 0995	(+)	(+)	(+)	(+)	(+)	(+)	(+)	(-)	(-)	(+)	(+)	(-)	(+)	(+)	(-)	(+)	(+)	(+)	(+)	(+)	Putative fumarylacetoacetate (FAA) hydrolase protein
	GDI 1003	(+)	(+)	(+)	(+)	(+)	(+)	(+)	(-)	(+)	(+)	(-)	(-)	(+)	(+)	(-)	(+)	(+)	(+)	(-)	(+)	Putative glutathione S-transferase
13	GDI 1053	(+)	(+)	(+)	(-)	(+)	(+)	(+)	(+)	(-)	(-)	(+)	(-)	(+)	(+)	(+)	(+)	(+)	(-)	(+)	(+)	Putative peptidase
	GDI 1106	(+)	(-)	(-)	(-)	(+)	(-)	(-)	(-)	(-)	(+)	(-)	(-)	(+)	(+)	(-)	(-)	(-)	(-)	(+)	(-)	Putative outer membrane factor
14	GDI 1564	(+)	(+)	(+)	(+)	(+)	(+)	(+)	(-)	(-)	(+)	(+)	(-)	(+)	(+)	(-)	(+)	(+)	(+)	(+)	(+)	Potassium-transporting ATPase B chain
	GDI 1566	(+)	(-)	(-)	(-)	(+)	(+)	(+)	(-)	(+)	(+)	(-)	(-)	(+)	(+)	(-)	(+)	(+)	(-)	(-)	(+)	Putative two-component sensor histidine kinase, sensor protein kdpD
15	GDI 1645	(+)	(+)	(+)	(+)	(+)	(+)	(+)	(+)	(+)	(+)	(+)	(+)	(+)	(+)	(+)	(+)	(+)	(+)	(+)	(+)	Putative flagellar biosynthesis protein flhA
	GDI 1662	(+)	(+)	(+)	(+)	(+)	(+)	(+)	(+)	(+)	(+)	(+)	(+)	(+)	(+)	(+)	(+)	(+)	(+)	(+)	(+)	Putative chemoreceptor mcpA (Methyl-accepting chemotaxis protein)
	GDI 1716	(+)	(+)	(+)	(+)	(+)	(+)	(-)	(+)	(+)	(+)	(+)	(+)	(+)	(+)	(+)	(+)	(+)	(+)	(+)	(+)	Putative amine oxidase
16	GDI 2134	(+)	(+)	(+)	(+)	(+)	(+)	(+)	(+)	(+)	(+)	(+)	(+)	(+)	(+)	(+)	(+)	(+)	(+)	(+)	(+)	GTP-binding protein engB
17	GDI 2248	(+)	(+)	(+)	(+)	(+)	(+)	(-)	(+)	(+)	(+)	(+)	(-)	(+)	(+)	(+)	(+)	(+)	(+)	(-)	(+)	Putative transcriptional regulator
18	GDI 2341	(+)	(+)	(+)	(+)	(+)	(+)	(+)	(+)	(+)	(+)	(+)	(+)	(+)	(+)	(+)	(+)	(+)	(+)	(+)	(+)	Putative N-acetylmuramoyl-L-alanine amidase amiA precursor
	GDI 2350	(+)	(+)	(+)	(+)	(+)	(+)	(+)	(+)	(+)	(+)	(+)	(+)	(+)	(+)	(+)	(+)	(+)	(+)	(+)	(+)	2-octaprenyl-6-methoxyphenol hydroxylase
19	GDI 2402	(+)	(+)	(+)	(+)	(+)	(+)	(+)	(-)	(+)	(+)	(+)	(+)	(+)	(+)	(+)	(+)	(+)	(+)	(+)	(+)	Putative capsular polysaccharide biosynthesis protein
	GDI 2419	(+)	(+)	(+)	(+)	(+)	(+)	(+)	(+)	(+)	(+)	(+)	(+)	(+)	(+)	(+)	(+)	(+)	(+)	(+)	(+)	Putative adenylate cyclase
20	GDI 2600	(+)	(-)	(+)	(-)	(+)	(-)	(-)	(-)	(+)	(+)	(-)	(-)	(+)	(+)	(-)	(+)	(+)	(-)	(-)	(+)	Putative sugar fermentation stimulation protein B
21	GDI 2695	(+)	(+)	(+)	(+)	(+)	(-)	(+)	(+)	(+)	(+)	(+)	(+)	(+)	(+)	(+)	(+)	(+)	(+)	(-)	(+)	Putative ribokinase
	GDI 2713	(-)	(-)	(+)	(-)	(-)	(-)	(-)	(+)	(+)	(+)	(+)	(-)	(+)	(+)	(-)	(+)	(+)	(-)	(-)	(-)	Putative opine oxidase subunit A
	GDI 2782	(+)	(-)	(+)	(-)	(-)	(-)	(-)	(-)	(+)	(+)	(-)	(+)	(+)	(-)	(-)	(-)	(-)	(-)	(-)	(+)	Putative H^+/Cl^- exchange transporter
	GDI 2833	(-)	(-)	(-)	(-)	(-)	(-)	(-)	(-)	(-)	(-)	(-)	(-)	(+)	(+)	(-)	(-)	(-)	(-)	(-)	(-)	Putative nonribosomal peptide synthetases (NPRS)
	GDI 2893	(-)	(+)	(-)	(-)	(-)	(-)	(-)	(-)	(-)	(-)	(-)	(-)	(+)	(+)	(-)	(-)	(+)	(-)	(-)	(-)	Putative patatin-like phospholipase
22	GDI 2987	(+)	(-)	(+)	(-)	(-)	(-)	(-)	(-)	(+)	(+)	(-)	(-)	(+)	(-)	(-)	(-)	(-)	(-)	(-)	(-)	Putative methyltransferase
	GDI 3005	(+)	(-)	(-)	(-)	(-)	(+)	(-)	(-)	(+)	(+)	(-)	(-)	(+)	(-)	(-)	(-)	(-)	(-)	(+)	(+)	Putative flavoprotein oxidoreductase
25	GDI 3627	(+)	(-)	(-)	(+)	(+)	(+)	(+)	(+)	(+)	(+)	(+)	(-)	(+)	(+)	(+)	(+)	(+)	(+)	(-)	(-)	Putative 3-carboxy-cis,cis-muconate cycloisomerase
28	GDI 3808	(+)	(+)	(+)	(-)	(-)	(+)	(-)	(-)	(+)	(+)	(-)	(+)	(+)	(+)	(-)	(+)	(+)	(-)	(+)	(+)	Putative tripeptide permease
Controls	GDI 0018	(+)	(+)	(+)	(+)	(+)	(+)	(+)	(-)	(+)	(+)	(+)	(+)	(+)	(+)	(+)	(+)	(+)	(+)	(+)	(+)	Nitrilase/cyanide hydratase
	GDI 0287	(+)	(+)	(+)	(+)	(+)	(+)	(+)	(+)	(+)	(+)	(+)	(+)	(+)	(+)	(+)	(+)	(+)	(+)	(+)	(+)	6-phosphogluconate dehydrogenase
	GDI 0429	(+)	(-)	(+)	(+)	(+)	(+)	(+)	(+)	(+)	(+)	(+)	(+)	(+)	(+)	(+)	(+)	(+)	(+)	(+)	(+)	Nif-specific regulatory protein NifA
	GDI 0491	(+)	(+)	(+)	(+)	(+)	(+)	(+)	(+)	(+)	(+)	(+)	(+)	(+)	(+)	(+)	(+)	(+)	(+)	(+)	(+)	Fructose-bisphosphate aldolase class I
	GDI 0525	(+)	(+)	(+)	(+)	(+)	(+)	(+)	(+)	(+)	(+)	(+)	(+)	(+)	(+)	(+)	(+)	(+)	(+)	(+)	(+)	Glutamine synthetase
	GDI 0548	(+)	(+)	(+)	(+)	(+)	(+)	(+)	(+)	(+)	(+)	(+)	(+)	(+)	(+)	(+)	(+)	(+)	(+)	(+)	(+)	Putative malto-oligosyltrehalose trehalohydrolase
	GDI 0694	(+)	(+)	(+)	(+)	(+)	(+)	(+)	(+)	(+)	(+)	(+)	(+)	(+)	(+)	(+)	(+)	(+)	(+)	(+)	(+)	ATP synthase subunit alpha
	GDI 0771	(+)	(+)	(+)	(+)	(+)	(+)	(+)	(+)	(+)	(+)	(+)	(+)	(+)	(+)	(+)	(+)	(+)	(+)	(+)	(+)	Molybdenum cofactor biosynthesis protein B
	GDI 0823	(+)	(+)	(+)	(+)	(+)	(+)	(+)	(+)	(+)	(+)	(+)	(+)	(+)	(+)	(+)	(+)	(+)	(+)	(+)	(+)	Dipeptidyl peptidase IV
	GDI 1183	(+)	(+)	(+)	(+)	(+)	(+)	(+)	(+)	(+)	(+)	(+)	(+)	(+)	(+)	(+)	(+)	(+)	(+)	(+)	(+)	Bifunctional purine biosynthesis protein purH
	GDI 1202	(+)	(+)	(+)	(+)	(+)	(+)	(+)	(+)	(+)	(+)	(+)	(+)	(+)	(+)	(+)	(+)	(+)	(+)	(+)	(+)	L-idonate 5-dehydrogenase
	GDI 1618	(+)	(+)	(+)	(+)	(+)	(+)	(+)	(+)	(+)	(+)	(+)	(+)	(+)	(+)	(+)	(+)	(+)	(+)	(+)	(+)	Acetate kinase
	GDI 1934	(+)	(+)	(+)	(+)	(+)	(+)	(+)	(+)	(+)	(+)	(+)	(+)	(+)	(+)	(+)	(+)	(+)	(+)	(+)	(+)	Anthranilate synthase component 1
	GDI 2062	(+)	(+)	(+)	(+)	(+)	(+)	(+)	(+)	(+)	(+)	(+)	(+)	(+)	(+)	(+)	(+)	(+)	(+)	(+)	(+)	Periplasmic binding protein
	GDI 2534	(+)	(+)	(+)	(+)	(+)	(+)	(+)	(+)	(+)	(+)	(+)	(+)	(+)	(+)	(+)	(+)	(+)	(+)	(+)	(+)	Putative transporter protein
	GDI 2623	(+)	(+)	(+)	(+)	(+)	(+)	(+)	(+)	(+)	(+)	(+)	(+)	(+)	(+)	(+)	(+)	(+)	(+)	(+)	(+)	Acetoin (diacetyl) reductase
	GDI 2625	(+)	(+)	(+)	(+)	(+)	(+)	(+)	(+)	(+)	(+)	(+)	(+)	(+)	(+)	(+)	(+)	(+)	(+)	(+)	(+)	Putative transporter protein

(+) PCR-positive
(-) PCR-negative
T: Type Strain
