# Supplementary figures and images for: Complete genome sequence of the sugarcane nitrogen-fixing endophyte Gluconacetobacter diazotrophicus Pal5
Source: BMC Genomics. 2009 Sep 23;10:450. doi: 10.1186/1471-2164-10-450 (PMC2765452; doi:10.1186/1471-2164-10-450)

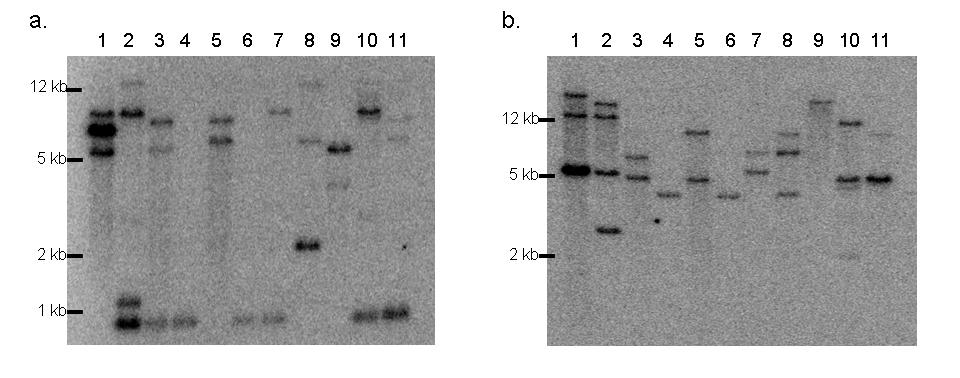

Supplement: Additional file 6 — Presence of homologues of the trbE gene among G. diazotrophicus strains. Total DNA of 11 Gluconacetobacter strains was completely digested with restriction enzymes EcoRI (a.) or EcoRV (b.), separated on agarose gel and submitted to Southern blot analysis using a fragment of CDS GDI0133 (trbE, part of type IV secretion system) as a probe. Numbers 1-10 represent G. diazotrophicus strains: Pal5 (1), 3R2 (2), URU (3), 38f2 (4), PRJ50(5), Pal3 (6), AF3 (7), PCRI (8), PPe4 (9), CNFe-550 (10). Number 11 represents G. johannae. In strain Pal5, only 3 bands are present, although the genome sequence indicates the presence of four copies of the trbE gene. However, the fourth trbE paralog (GDI1016) is more dissimilar to the probe sequence then the other three (GDI0133, GDI2742 e GDI2911), which may have prevented hybridization. [file 1471-2164-10-450-S6.JPEG]
