# Supplementary material for: Complete genome sequence of the sugarcane nitrogen-fixing endophyte Gluconacetobacter diazotrophicus Pal5
Source: BMC Genomics. 2009 Sep 23;10:450. doi: 10.1186/1471-2164-10-450 (PMC2765452; doi:10.1186/1471-2164-10-450)
Supplement: Additional file 8 — Number of Reciprocal Best Hits (RBH) in accessory and core regions. The first column shows the number of RBH for each organism in parentheses. The RBH in columns show the total number of RBH for each organism. The RBH % by organism columns shows the percent of RBH in relation with the total number of RBH found in accessory and core regions. RBH result has 708 RBH in accessory regions and 2,258 in core regions. The RBH % by organism columns shows the percentage of RBH in accessory and core regions for each organism or group. GOX = Gluconobacter oxydans 621H, GBE = Granulibacter bethesdensis CGDNIH, ACR = Acidiphilium cryptum JF-5, Rhiz = All the complete genomes from Rhizobiales order, Other Alpha = All other complete genomes from Alphaproteobacteria class, Beta = All complete genomes from Betaproteobacteria class, Gamma = All complete genomes from Gammaproteobacteria class, Others = All other complete genomes. [file 1471-2164-10-450-S8.PDF]

Groups	RBH in accessory	RBH in core	RBH % in accessory (708)	RBH % in core (2,258)	RBH % by organism accessory	RBH % by organism core
GOX (1,099)	113	986	16%	44%	10%	90%
GBE (337)	25	312	3.5%	14%	7%	93%
ACR (137)	11	126	1.5%	5.6%	8%	92%
Rhiz (643)	283	360	40%	16%	44%	56%
Other Alpha (254)	111	143	16%	6%	43%	56%
Beta (190)	57	133	8%	6%	30%	70%
Gamma (188)	67	121	9.5%	5.4%	36%	64%
Others (118)	41	77	6%	3.4%	35%	65%
