# Supplementary material for: Complete genome sequence of the sugarcane nitrogen-fixing endophyte Gluconacetobacter diazotrophicus Pal5
Source: BMC Genomics. 2009 Sep 23;10:450. doi: 10.1186/1471-2164-10-450 (PMC2765452; doi:10.1186/1471-2164-10-450)
Supplement: Additional file 11 — Comparison of main signalling protein categories. AT, Agrobacterium tumefaciens C58; BJ, Bradyrhizobium japonicum USDA110; ML, Mesorhizobium loti MAFF303099, SM, Sinorhizobium meliloti 1021, GO, Gluconobacter oxydans 621H; RP, Rickettsia prowazekii MadridE; AB, Azoarcus sp. BH72; AE, Azoarcus sp. EbN1; XF, Xylella fastidiosa 9a5c; EC, Escherichia coli K12-MG1655. [file 1471-2164-10-450-S11.PDF]

	GD	AT	BJ	ML	SM	GO	RP	AB	AE	XF	EC
**GENOME SIZE (Mb)**	3,99	5,65	9,11	7,60	6,80	2,92	1,11	4,40	4,73	2,73	4,60
**TOTAL CDSs**											
**SIGNALLING CATEGORY**	3,852	5,360	8,317	7,272	6,224	2,664	835	3,989	4,603	2,832	4,243
**Kinases/phoshatase**											
Total CDSs	23	43	73	45	35	13	5	71	43	14	28
% in relation to total CDSs	0.60	0.80	0.88	0.62	0.56	0.49	0.60	1.78	0.93	0.49	0.66
% in relation to signaling CDSs	29.5	30.7	29.9	35.2	26.7	37.1	50.0	34.6	37.7	45.2	36.4
**Chemotaxis (MCP/methylesterase)**											
Total CDSs	11	21	37	2	13	4	0	27	4	2	6
% in relation to total CDSs	0.29	0.39	0.45	0.03	0.21	0.15	0	0.68	0.09	0.07	0.14
% in relation to signaling CDSs	14.1	15.0	15.2	1.56	9.92	11.4	0.00	13.2	3.51	6.45	7.79
**Second messenger (cAMP, cGMP)**											
Total CDSs	18	36	68	44	43	6	2	58	30	3	21
% in relation to total CDSs	0.47	0.67	0.82	0.61	0.69	0.23	0.24	1.45	0.65	0.11	0.50
% in relation to signaling CDSs	23.1	25.7	27.9	34.4	32.8	17.1	20.0	28.3	26.3	9.68	27.3
**Response regulators**											
Total CDSs	31	50	110	49	49	16	4	71	51	19	26
% in relation to total CDSs	0.81	0.93	1.32	0.67	0.79	0.60	0.48	1.78	1.11	0.67	0.61
% in relation to signaling CDSs	39.7	35.7	45.1	38.3	37.4	45.7	40.0	34.6	44.7	61.3	33.8
**Total**											
Total CDSs	78	140	244	128	131	35	10	205	114	31	77
% in relation to total CDSs	2.03	2.61	2.93	1.76	2.11	1.31	1.20	5.14	2.48	1.10	1.82
**NUMBER OF SIGNALLING CDSs PER Mb GENOME**	19.6	24.8	26.8	16.8	19.3	12.0	9.01	46.6	24.1	11.4	16.7
