# Supplementary material for: Complete genome sequence of the sugarcane nitrogen-fixing endophyte Gluconacetobacter diazotrophicus Pal5
Source: BMC Genomics. 2009 Sep 23;10:450. doi: 10.1186/1471-2164-10-450 (PMC2765452; doi:10.1186/1471-2164-10-450)
Supplement: Additional file 12 — Comparison of main transport-related protein categories. AT, Agrobacterium tumefaciens C58; BJ, Bradyrhizobium japonicum USDA110; ML, Mesorhizobium loti MAFF303099, SM, Sinorhizobium meliloti 1021, GO, Gluconobacter oxydans 621H; RP, Rickettsia prowazekii MadridE; AB, Azoarcus sp. BH72; AE, Azoarcus sp. BH72, XF, Xylella fastidiosa 9a5c; EC, Escherichia coli K12-MG1655. [file 1471-2164-10-450-S12.PDF]

	GD	AT	BJ	ML	SM	GO	RP	AB	AE	XF	EC
**GENOME SIZE (Mb)**	3,99	5,65	9,11	7,60	6,80	2,92	1,11	4,40	4,73	2,73	4,60
**TOTAL CDSs**	3,852	5,360	8,317	7,272	6,224	2,664	835	3,989	4,603	2,832	4,243
**TRANSPORT CATEGORY**											
**Primary active transporters**											
Total CDSs	257	694	653	671	611	104	26	100	147	58	225
% in relation to total CDSs	6.67	12.95	7.85	9.23	9.82	3.90	3.11	2.50	3.19	2.05	5.30
% in relation to transport CDSs	45.6	76.18	66.3	75.8	73.3	49.5	44.8	68.5	56.5	52.7	42.1
**Electrochemical potential-driven transporters**											
Total CDSs	167	198	310	182	198	92	30	41	97	39	244
% in relation to total CDSs	4.33	3.69	3.73	2.50	3.18	3.45	3.59	1.02	2.11	1.38	5.75
% in relation to transport CDSs	29.7	21.73	31.5	20.6	23.8	43.8	51.7	28.1	37.3	35.4	45.7
**Pores and channels**											
Total CDSs	68	12	12	19	17	10	1	2	11	7	13
% in relation to total CDSs	1.76	0.22	0.14	0.26	0.27	0.37	0.12	0.05	0.24	0.25	0.31
% in relation to transport CDSs	12.1	1.32	1.22	2.14	2.04	4.76	1.72	1.37	4.23	6.36	2.43
**Others**											
Total CDSs	71	7	10	13	7	4	1	3	5	6	52
% in relation to total CDSs	1.84	0.13	0.12	0.18	0.11	0.15	0.12	0.07	0.11	0.21	1.22
% in relation to transport CDSs	12.6	0.77	1.01	1.47	0.84	1.90	1.72	2.05	1.92	5.45	9.74
**Total**											
Total CDSs	563	911	985	885	833	210	58	146	260	110	534
% in relation to total CDSs	14.6	17.0	11.8	12.2	13.4	7.9	6.9	3.7	5.6	3.9	12.6
**NUMBER OF TRANSPORTERS PER Mb GENOME**	141.1	161.2	108.1	116.4	122.5	71.9	52.2	33.2	54.9	40.3	116.1
