# Supplementary material for: Complete genome sequence of the sugarcane nitrogen-fixing endophyte Gluconacetobacter diazotrophicus Pal5
Source: BMC Genomics. 2009 Sep 23;10:450. doi: 10.1186/1471-2164-10-450 (PMC2765452; doi:10.1186/1471-2164-10-450)
Supplement: Additional file 13 — Comparison among the two Gluconacetobacter diazotrophicus Pal5 genomic sequences. GDI-BR, NCBI RefSeq NC_010125, GDI-US, NCBI RefSeq NC_011365, GIs, Genome Islands. [file 1471-2164-10-450-S13.PDF]

**OVERVIEW**	**GDI-BR**	**GDI-US**
Total CDS in chromossome	3781	3472
Blast Best Hits BR x US	3034	3034
Percent of Blast Best Hits	80%	87%
Unique genes	747	438
Possible missing annotation	123	328
**PROFILE OF UNIQUE GENES**	**GDI-BR**	**GDI-US**
Hypothetical proteins	402	184
Transposases	102	76
Integrases	35	21

**LOCATION**	**Inside GIs**	**Outside GIs**
Blast Best Hits CDS	450 (15%)	2584 (85%)
Unique CDS GDI-BR	499 (67%)	248 (33%)
